# Genomic and functional analysis of stress-responsive prophages in *Lactobacillus helveticus*

**DOI:** 10.3389/fmicb.2026.1819103

**Published:** 2026-04-23

**Authors:** Andrea Mancini, Alessandro Cestaro, Paolo Sonego, Stefano Piazza, Nicola Cologna, Elena Franciosi

**Affiliations:** 1 Research and Innovation Centre, Fondazione Edmund Mach, San Michele all’Adige, Trento, Italy; 2 Trentingrana Consorzio dei Caseifici Sociali Trentini s.c.a., Trento, Italy

**Keywords:** *Caudoviricetes*, hard cheese, lactic acid, *Lactobacillus helveticus*, natural whey starter, tailed phage, temperate phage

## Abstract

**Introduction:**

Bacteriophages are pervasive components of natural whey starters (NWS) used in traditional hard cheese production, yet their functional role under cheesemaking-related stresses remains poorly understood.

**Methods:**

In this study, six temperate bacteriophages associated with *Lactobacillus helveticus* were isolated from Trentingrana NWS. Classified within the *Caudoviricetes* class, they harbored gene sets associated with the typical phage cycle together with a large number of open reading frames (ORFs) with hypothetical or unknown function. By combining quantitative PCR targeting hallmark phage genes with detection of extracellular phage particles, we investigated prophage induction dynamics under abiotic conditions relevant to dairy processing, including heat shock, nutrient limitation, lactic acid, and oxidative stress.

**Results:**

Across conditions, stress exposure elicited phage-specific and dose-dependent responses, frequently revealing a decoupling between early increases in phage genomic copy number and subsequent virion release. Heat shock emerged as the most consistent inducer of prophage activation, whereas lactic acid and oxidative stress produced threshold-dependent or inhibitory effects, particularly at higher intensities. Nutrient limitation alone had limited impact, suggesting a permissive rather than triggering role.

**Discussion:**

Overall, these findings support a non-binary model of prophage induction, spanning intermediate states from partial genome replication to productive lytic cycles, strongly constrained by host physiological status. Our results indicate that *L. helveticus* prophages respond heterogeneously to cheesemaking-related stresses and may act as context-dependent modulators of NWS stability rather than direct disruptors. Such stress-responsive prophage dynamics are likely to influence microbial population turnover and resilience during back-slopping, ultimately contributing to the long-term stability and functional robustness of Trentingrana natural whey starters.

## 1 Introduction

As obligate intracellular parasites, bacteriophages, also known as phages, inhabit all the ecosystems where bacteria exist, including anthropogenic environments housing both selected and unselected bacterial populations (Carminati et al., 2010; Mahony et al., 2016; Queiroz et al., 2022; Somerville et al., 2022; Ortiz Charneco et al., 2023).

The presence of phages in these human-influenced environments has a well-documented history, posing a recognized challenge in the dairy plant. Indeed, phage attack is associated with loss of fitness in specific industrial processes, such as milk fermentation (Jolicoeur et al., 2023; Ortiz Charneco et al., 2023), propagation and maintenance of probiotic strains and defined or undefined starter cultures (Pei et al., 2021; Wu et al., 2023; Kamiński and Paczesny, 2024) and, as a result, in cheese making (Paillet et al., 2022, 2024; Somerville et al., 2022; Decadt et al., 2023). In these environments, each phage-bacteria interaction is characterized by high strain specificity (Mahony et al., 2016; Queiroz et al., 2022), which, depending on the dairy process and the bacteria consortium, also drives a particular viral presence (Somerville et al., 2022, 2024; Ortiz Charneco et al., 2023). A next-generation sequencing (NGS) study of artisanal raw milk cheese has shown the constant interaction between phage and bacterial hosts, from the NWS to the cheese, and differences were observed among dairies (Queiroz et al., 2022). Moreover, traces of interaction between phage-bacterial hosts were represented by the presence of specific anti-phage defense mechanisms in the metagenome-assembled genomes (MAGs), particularly defense genes belonging to RM, Abi, and CRISPR-Cas type I, II, and III (Queiroz et al., 2022; Somerville et al., 2022, 2024). However, it is also known that the effect of bacteriophages in perturbing the microbial ecology is context-dependent, with the same phage being beneficial or costly depending on subtle behavioral changes or the surrounding bacterial community (Thomas et al., 2024).

The majority of known bacteriophages are temperate phages, or phages able to enter a quiescent state by integrating into the bacterial host genome (Brady et al., 2021). The switch between the lysogenic and lytic cycles, with consequent release of phage particles, has major ecological significance: while lytic infections rapidly alter community structure and nutrient flow via the viral shunt, lysogeny exerts more complex, long-term effects (Zhang et al., 2022). Particularly, prophage induction strongly shapes bacterial community dynamics by regulating population abundance according to the Kill-the-Winner model (Huang et al., 2024), limiting dominant taxa and maintaining community diversity. Although induction leads to the loss of individual lysogens, this cost is offset at the population level by reduced cell density and increased availability of resources (Zhang et al., 2022). Experimental validation shows that lysis probability is a key determinant of phage effectiveness, while nutrient availability emerges as an additional critical factor shaping lysogen dynamics (Thomas et al., 2024). Temperate bacteriophages may also confer superinfection exclusion to their lysogenic hosts, allowing lysogens to use phages as self-amplifying weapons against susceptible competitors. In these conditions, low-level spontaneous induction releases phage particles that infect and lyse nearby susceptible bacteria, enhancing the ability of lysogens to invade and defend ecological niches (Thomas et al., 2024).

Traditional food fermentations rely on spontaneous microbiota from raw materials, leading to variable outcomes. In this context, back-slopping or the reuse of a portion of a successful fermentation as inoculum is a well-established and used methodology that improves process reliability and guides microbial communities toward optimal composition. Indeed, over time, this practice enables the independent evolution of complex microbial ecosystems shaped by population dynamics, strain evolution, and environmental inputs (Neviani, 2024). Trentingrana, a Protected Designation of Origin (PDO) hard Grana-like cheese produced in Northern Italy (Franciosi et al., 2012; Bertani et al., 2020; Mancini et al., 2021), is characterized by the use of a NWS culture in the cheese-making process that greatly influences the peculiarity and organoleptic characteristics of this cheese (Gobbetti et al., 2018; Bertani et al., 2020; Neviani et al., 2013). Daily maintained in the dairy plant with a back-slopping approach, this starter comes from the whey collected from the vat after curd precipitation, and it is mainly characterized by thermophilic lactic acid bacteria (LAB) such as *Lactobacillus helveticus*, *Lactobacillus delbrueckii* ssp. *bulgaricus* and *Lactobacillus delbrueckii* ssp. *lactis* (Rossetti and Giraffa, 2005; Neviani et al., 2013). The presence of phages in NWSs has the potential to lead to a decline in the acidification activity, consequently giving rise to several associated issues that could detrimentally impact the quality or the yield of the final product. This problem may include slow and/or incomplete fermentation, incomplete whey purge from the curd, growth of pathogenic or spoilage bacteria, all factors that negatively affect the quality or the yield of the final product (Mancini et al., 2021).

With the advancement and widespread adoption of genome sequencing, there is an increasing need for a deeper understanding of the genomic traits of phages infecting lactobacilli, particularly within microbial consortia such as those found in NWS. Only a limited number of studies have delved into the characterization of phages derived from Grana-like cheeses, documenting the coexistence of phages, particularly with *Lactobacillus helveticus* and *Lactobacillus delbrueckii* ssp. (Zago et al., 2006, 2008, 2013, 2015; Giraffa et al., 2017; Mancini et al., 2021; Queiroz et al., 2022). In this context, the genome of *Lactobacillus helveticus* phage ϕAQ113 (NC_019782.1) established an important reference for Grana-associated dairy phages, while surveys of Trentingrana NWS revealed widespread phage occurrence across dairies and time points, with links to *L. helveticus* population structure but no simple correspondence with loss of acidification performance (Zago et al., 2013; Mancini et al., 2021). More broadly, recent studies on lactobacilli and artisanal cheese microbiomes indicate that prophages are widespread and that phage-host interactions in undefined starter communities can be stable, persistent, and ecologically consequential rather than purely disruptive (Mancini et al., 2021; Somerville et al., 2022). Against this background, what remains unclear is how prophages carried by dominant NWS bacteria respond to the abiotic stresses that repeatedly arise during back-slopping.

Here, using the artisanal Trentingrana NWS system as a naturally informative model, we combined phage genomics, host range, morphological, and comparative analyses with stress-induction assays on six temperate *Lactobacillus helveticus* phages to test whether cheesemaking-relevant abiotic cues that may arise at the interface of the back-slopping process could trigger uniform productive induction or phage-specific intermediate activation states. Our findings underscore the importance of investigating the genetic features of dairy phage to elucidate phage-bacteria interactions and their role in cheese-making production.

## 2 Materials and methods

### 2.1 Bacterial strains and cultivation media

Phage bacterial hosts were isolated from NWS samples collected over 1 year of Trentingrana production in five dairy plants belonging to the Trentingrana Consortium, as reported in Mancini et al. (2021). All the bacterial strains, identified as *Lactobacillus helveticus*, were maintained from −80 °C stocks in modified Man, Rogosa and Sharpe (MMRS) broth by the addition to the MRS media of yeast extract (40 g/L), tween 80 (1 mL/L), acidified to pH 5.8 using a 5 M lactic acid solution. These strains were grown at 45 °C under anaerobic conditions for 24 h. Culture media and anaerobic systems were purchased from Oxoid (Thermo Fisher Scientific, Milan, Italy).

### 2.2 Phage isolation

The six phages studied herein (ϕCR191, ϕP185, ϕCV244, ϕS16, ϕS193, and ϕT280) were isolated from NWS-derived *Lactobacillus helveticus* strains by means of Mitomycin C (MC) induction. Particularly, we selected six *L. helveticus* strains (namely CR191, P185, CV244, S16, S193, and T280) belonging to the most conserved biotype and characterized by strong acidification activity in experimental whey (data not shown). Two tubes with MMRS broth were prepared: the first tube contained 2% (vol/vol) of *L. helveticus* broth culture in the logarithmic growth phase supplemented with MC at [1 μg/mL]; the second tube contained 2% (vol/vol) of *L. helveticus* broth culture in the logarithmic growth phase and was used as the untreated control. The tubes were incubated at 45 °C until cell lysis occurred, as the clearing of the MC added broth, compared to the control tube. When lysis was observed, the tubes were filtered through a 0.45-μm pore-size membrane filter (Merck–Millipore, Darmstadt, Germany), pH adjusted to 7, and the solution stored at 4 °C until use, for no more than 7 days. Phage stocks were stored at −80 °C with 40% vol/vol of glycerol.

### 2.3 Transmission electron microscopy

Phages were concentrated from a 50 mL phage lysate obtained with the respective *L. helveticus* cell host and prepared for transmission electron microscopy (TEM) as follows: bacterial cells were removed by centrifugation at 4,000 g for 10 min, then lysate solution was filtered through a 0.45-μm membrane filter pore size (Merck–Millipore, Darmstadt, Germany), and 0.5 M of NaCl and PEG 6000 (final concentration of 10%) were added and mixed until complete solution. Tubes were then stored at 4 °C overnight. Phages were subsequently collected by centrifugation at 10,000 g for 15 min; resuspended in 500 μL of TMG buffer solution (Tris–HCl 10 mM, pH 7.7; MgSO_4_ 10 mM), then stored at 4 °C overnight. Final phage preparation was obtained by centrifugation at 9,000 g for 15 min, discarding the pellet constituted by PEG, and a new centrifugation of the supernatant at 14,000 g for 30 min. Phage pellets were resuspended in 100 μL of TMG solution with the addition of NaCl 0.5 M.

Samples for electron microscopy were prepared by adding 10 μL of phage suspension over a carbon-coated copper grid, then stained with 1% (w/v) phosphotungstic acid (pH 6.5) (Sigma-Aldrich, Milan, Italy) solution for 10 min, and images captured at C. I. G. S. - Centro Interdipartimentale Grandi Strumenti Università di Modena e Reggio Emilia (Modena, Italy) with a Talos F200S G2 transmission electron microscope (Thermo Fisher Scientific) at 200 kV.

### 2.4 Phages’ host range determination

Titer and the lytic spectrum were determined by plaque lysis assay as reported in Mancini et al. (2021) with some modification. Briefly, 392 bacterial strains previously isolated from the same Trentingrana NWSs and identified as *L. helveticus* (Mancini et al., 2021), were randomized (through www.random.org), and the first 60 were used for the test. The six *L. helveticus* host strains of the isolated bacteriophages were also included in this assay, for a total of 67 *L. helveticus* strains. Then 10 μL of 10^−1^ phage dilution (initial PFU/mL titer ranging from 8 × 10^4^ to 4 × 10^5^) were poured on MMRS-Ca (MMRS with 10 mM of CaCl_2_) agar media containing selected bacterial strains, and after incubation at 45 °C for 24 h in anaerobic conditions, cell lysis was checked with a degree considered as complete (+++), partial (++), low (+) or absent (−) (Table 1).

**Table 1:** Host range of the six *Lactobacillus helveticus* phages considered in this study.

Strain	Dairy implant	Period of isolation	Phage					
			ϕCR191	ϕCV244	ϕP185	ϕS16	ϕS193	ϕT280
*L. helveticus*_CR62	CR	February	−	−	−	−	−	−
*L. helveticus*_CR67	CR	February	−	−	−	−	−	−
*L. helveticus*_CR72	CR	February	−	−	−	−	−	−
*L. helveticus*_CR80	CR	February	−	−	−	−	−	−
*L. helveticus*_CR81	CR	February	−	−	−	−	−	−
*L. helveticus*_CR93	CR	May	−	−	−	−	−	−
*L. helveticus*_CR100	CR	May	−	−	−	−	−	−
*L. helveticus*_CR102	CR	May	−	−	−	−	−	−
*L. helveticus*_CR104	CR	May	−	−	−	−	−	−
*L. helveticus*_CR106	CR	September	−	−	−	−	−	−
*L. helveticus*_CR108	CR	September	−	−	−	−	−	−
*L. helveticus*_CR119	CR	December	−	−	−	−	−	−
*L. helveticus*_CR113	CR	December	−	−	−	−	−	−
*L. helveticus*_CV151	CV	February	−	−	−	−	−	−
*L. helveticus*_CV164	CV	September	−	−	−	−	−	−
*L. helveticus*_CV170	CV	September	−	−	−	−	−	−
*L. helveticus*_CV349	CV	December	−	−	−	−	−	−
*L. helveticus*_P86	P	May	−	−	−	−	−	−
*L. helveticus*_P135	P	May	−	−	−	−	−	−
*L. helveticus*_P165	P	September	−	−	−	−	−	−
*L. helveticus*_P169	P	September	−	−	−	−	−	−
*L. helveticus*_P223	P	September	−	−	−	−	−	−
*L. helveticus*_P242	P	September	−	−	−	−	−	−
*L. helveticus*_P258	P	September	−	−	−	−	−	−
*L. helveticus*_P273	P	December	−	−	−	−	−	−
*L. helveticus*_P302	P	December	−	−	−	−	−	−
*L. helveticus*_P315	P	December	−	−	−	−	−	−
*L. helveticus*_P317	P	December	−	−	−	−	−	−
*L. helveticus*_P343	P	December	−	−	−	−	−	−
*L. helveticus*_R66	R	May	−	−	−	−	−	−
*L. helveticus*_R81	R	May	−	+	+	−	−	−
*L. helveticus*_R94	R	May	−	−	−	−	−	−
*L. helveticus*_R96	R	May	−	−	−	−	−	−
*L. helveticus*_R102	R	May	−	−	−	−	−	−
*L. helveticus*_R128	R	May	−	−	−	−	−	−
*L. helveticus*_R148	R	May	−	−	−	−	−	−
*L. helveticus*_R201	R	September	−	−	−	−	−	−
*L. helveticus*_R218	R	September	−	−	−	−	−	−
*L. helveticus*_R247	R	September	−	−	−	−	−	−
*L. helveticus*_R290	R	December	−	−	−	−	−	−
*L. helveticus*_R318	R	December	−	−	−	−	−	−
*L. helveticus*_S1	S	February	−	−	−	−	−	−
*L. helveticus*_S3	S	February	−	−	−	−	−	−
*L. helveticus*_S28	S	February	−	−	−	−	−	−
*L. helveticus*_S40	S	February	−	−	−	−	−	−
*L. helveticus*_S66	S	February	−	−	−	−	−	−
*L. helveticus*_S200	S	September	−	−	−	−	−	−
*L. helveticus*_S247	S	September	−	−	−	−	−	−
*L. helveticus*_S271	S	December	−	−	−	−	−	−
*L. helveticus*_S273	S	December	−	−	−	−	−	−
*L. helveticus*_S276	S	December	−	−	−	−	−	−
*L. helveticus*_S312	S	December	−	−	−	−	−	−
*L. helveticus*_S346	S	December	−	−	−	−	−	−
*L. helveticus*_S348	S	December	−	−	−	−	−	−
*L. helveticus*_T173	T	September	−	−	−	−	−	−
*L. helveticus*_T195	T	September	−	−	−	−	−	−
*L. helveticus*_T221	T	September	+	+	+	+	+	++
*L. helveticus*_T260	T	December	−	−	−	−	−	−
*L. helveticus*_CR28	CR	September	−	−	−	−	−	−
*L. helveticus*_CR191	CR	September	+++	−	−	−	−	−
*L. helveticus*_CV244	CV	September	−	+++	−	−	−	−
*L. helveticus*_P185	P	September	−	−	+++	−	−	−
*L. helveticus*_S16	S	February	−	−	−	+++	−	−
*L. helveticus*_S193	S	May	−	−	−	−	+++	−
*L. helveticus*_T280	T	December	−	−	−	−	−	+++

Strains’ susceptibility to phage infection is indicated as degree of “−” to “+++.” “+++” In heavy grey cells. “+++” indicates a completely clear zone in grey cells. “++” indicates a center clear zone with a blurred ring. “+” indicates a generally clear zone. In light grey cells “−” “indicates no; black cells refer to complete lysis in the corresponding host strains. Dairy implants: T, western area of the Trentino province; CV, north-western area of the Trentino province; R, north-western area of the Trentino province; CR, northern area of the Trentino province; P, eastern area of the Trentino province; S, southern area of the Trentino province.

### 2.5 Phage DNA extraction

Phage DNA was extracted by means of a phenol-chloroform method: 50 mL phage lysate obtained with the respective *L. helveticus* host strain were centrifuged at 4,000 g for 10 min at 4 °C, filtered through a 0.45-μm membrane filter pore size (Merck–Millipore), with the addition of 0.5 M NaCl and PEG 6000 (final concentration of 10%), and gently mixed until complete solution. Tubes were stored at 4 °C overnight. Phages were subsequently collected by centrifugation at 10,000 g for 15 min at 4 °C. Pellets were resuspended in 1 mL of cold SM buffer (200 mM NaCl, 10 mM MgSO4, 50 mM Tris–HCl, pH 7.5), with the addition of 2 U DNase (Invitrogen, Thermo Fisher Scientific, Milan, Italy). After incubation at 37 °C for 30 min, 10 μL 0.5 M EDTA was added, and the pellets were stored on ice for 5 min before the beginning of solvents steps. Tubes were added with one volume of Tris-saturated phenol (Thermo Fisher Scientific, Milan, Italy), mixed by vortex, and centrifuged at 8,500 g for 10 min at 4 °C. This step was repeated twice. After the addition of another volume of chloroform:isoamilic alcohol solution, the tubes were mixed by vortex and centrifuged at 7,000 g for 10 min at 4 °C. DNA was precipitated by the addition of one volume of cold isoamyl alcohol and 50 μL of sodium acetate (3 M, pH 5.2), and samples were stored at 4 °C overnight. DNAs were collected by centrifugation at 10,000 g for 10 min at 4 °C, and rinsed with 800 μL of cold 70% ethanol, discarded after centrifugation at 10,000 g for 10 min at 4 °C. The DNA pellet was finally resuspended in 30 μL of TE buffer (10 mM Tris–HCl, 1 mM EDTA, pH 7.6).

DNAs were checked for quality and quantity with NanoDropTM 8,000 Microvolume UV–Vis spectrophotometer (Thermo Fisher Scientific, Milan, Italy), while the integrity of DNAs was verified by agarose gel electrophoresis.

### 2.6 Genome sequencing, annotation, and analysis

Genomic DNA from each phage was prepared for sequencing on an Oxford Nanopore Technologies device^1^ using PCR Barcoding Expansion 96 (EXP-PCB096) and Ligation Sequencing kit (SQK-LSK109), following the manufacturer’s protocol. Sequencing was run using Flowcell version R10.3 (FLO-MIN111; Oxford Nanopore Technologies) for a 72 h running time.

Guppy v 5.0.14^2^ was used to perform base calling to convert MinION raw FAST5 outputs to FASTQ files. The raw reads in the FASTQ files were filtered for quality using Filtlong v0.2.1.^3^ Filtered reads were assembled using Flye v2.9.0 (Kolmogorov et al., 2019) and polished by Medaka v1.4.4.^4^ For all software, default parameters were used unless otherwise noted. The draft genome sizes ranged from 31.5 to 39.5 kb, with the GC content ranging from 35.8 to 39.3%. Genome sequences have been deposited in the NCBI GenBank database with the following accession numbers: OQ627803.1, OQ627804.1, OQ627805.1, OQ627806.1, OQ627807.1, OQ627808.1. Sequence metrics are listed in Table 2.

**Table 2:** Genome and features information of phages considered in this study.

Name	Genome size (kbp)	Contig	GC content (%)	ORFs	Capside size	Tail size	BLASTn	GenBank
					(nm)	(nm)	(%identity hit, query cover)	Accession members
CR191	35.411	1	37.45	68	61.9 ± 0.2	140.8 ± 0.2	97.07%-ϕAQ113.	OQ627803.1
							57%	
CV244	39.435	1	39.31	74	56.8 ± 0.2	273.7 ± 0.2	96.02%-ϕAQ113.	OQ627804.1
							10%	
P185	39.321	1	36.41	80	61.3 ± 0.2	159.5 ± 0.2	95.29%-ϕAQ113.	OQ627805.1
							55%	
S16	31.477	1	36.36	77	71.2 ± 0.2	94.8 ± 0.2	95.74%-ϕAQ113.	OQ627806.1
							58%	
S193	36.440	1	35.79	64	88.4 ± 0.2	147.9 ± 0.2	95.77%-ϕAQ113.	OQ627807.1
							57%	
T280	35.926	1	37.30	70	75.3 ± 0.2	99.5 ± 0.2	98.62%-ϕAQ113.	OQ627808.1
							50%	

Open reading frames (ORFs) were detected using PHANOTATE,^5^ while gene annotation was performed using PHAROKKA^6^ (Bouras et al., 2023), coupled with manual annotation against the UniProt database where needed (The UniProt Consortium et al., 2023). Presence of auxiliary metabolic genes (AMGs) was detected using DARM-v (Shaffer et al., 2020), while the temperateness of the considered phage was confirmed through VIBRANT (Kieft et al., 2020).

Protein functional annotation was further refined using InterProScan (Jones et al., 2014), implemented through the Galaxy Europe platform,^7^ to identify conserved domains and assign putative functions to regulatory proteins. Regulatory genes of interest were extracted and aligned using MAFFT (Katoh and Standley, 2013), also via Galaxy, and the resulting multiple sequence alignments were used for phylogenetic reconstruction as described above. Comparative genome organization and synteny were visualized using clinker/clustermap.js (Gilchrist and Chooi, 2021) and phylogeny-related results with iTOL (Letunic and Bork, 2024).

To assess genome-level relatedness and clustering, gene-sharing network analysis was performed using vConTACT2 (Jang et al., 2019) through the Galaxy platform. Network outputs were visualized and explored using Cytoscape (Shannon et al., 2003).

ViPTree web server^8^ (Nishimura et al., 2017) with database updated to November 2023 was used to evaluate the taxonomic affiliation of our phages at the subfamily or family level; genome comparisons were visualized using LoVis4U (Egorov and Atkinson, 2025), while genome maps were generated using SnapGene software.^9^

### 2.7 Prophage induction

The temperate lifestyle of each phage was investigated in its corresponding *L. helveticus* phage host. Each bacterial strain was grown at 45 °C without agitation, by inoculating at 2% (vol/vol) 50 mL MMRS medium. Then, in early exponential phase (OD 600 nm–0.2), the following conditions were tested: (i) bacterial inoculum (or control, ctrl); (ii) MC [1 μg/mL]; (iii) H_2_O_2_ at 0.2 and 0.4 mM; (iv) lactic acid (LA) at 0.2 and 0.8%; (v) heat shock at 65 °C for 2 h; (vi) nutrient limitation as 1:2 diluted MMRS broth media. After 2 h 2 mL aliquot was collected from each condition and placed at −80 °C for the subsequent phenol-chloroform DNA extraction. Bacterial growth was monitored for 24 h after the inoculum, and OD 600 nm reading growth profiles were finally generated.

To assess the presence of potentially induced prophage in supernatants, after 4 h from the inoculum, 30 mL of supernatants for each condition tested were collected, cell debris removed by centrifugation at 4,000 g for 10 min at 4 °C, and SN filtered through a 0.45-μm pore-size membrane filter (Merck–Millipore). Phage particles were then collected by adding PEG 6000 (final concentration of 10%), centrifuging at 10,000 g for 15 min at 4 °C, and finally resuspending the pellets in 1 mL of cold SM buffer. Subsequently, by means of plaque lysis assay, 100 μL of the bacterial host in the logarithmic growth phase was added to 5 mL of semisolid MMRS-Ca (4% wt/vol agar), and the mixture was poured on the surface of MMRS-Ca plates. Afterward, 10 μL of concentrated phages from SN were spotted onto the lawn of bacteria. After anaerobic incubation at 45 °C for 24 h, the lysis zone was checked. Experiments were carried out in triplicate.

### 2.8 Real-time PCR

Real-time PCR was carried out in 20 μL reactions containing 10 μL of 2x qPCRBIO SyGreen Mix Separate-ROX (PCR BioSystems, UK), 0.4 μL of each primer (10 ng/μL) (Supplementary Table S1), 5.2 μL of DEPC-treated water, and 4 μL of DNA (10 ng). Reactions were carried out in a LightCycler 480 (Roche, LifeScience, Italy) under the following conditions: 95 °C for 15 s, followed by 45 cycles at 95 °C for 15 s, 63 °C for 30 s, 72 °C for 10 s, one cycle at 95 °C for 10 s, 65 °C for 15 s, 97 °C continuous and a final step at 40 °C for 30 s. CT values for each sample for baseplate wedge subunit (BWS), tail sheath (TS), endolysin (END), and minor head protein (MHP, only for phage ϕCV244) were normalized to the bacterial housekeeping gene *tuf* using the ΔCt, and data were shown as log2 fold changes derived from the ΔΔCt method. The RT-PCR was performed in duplicates for each DNA sample, and independent experiments were carried out in triplicate. Please refer to Supplementary Table S1 for the list of utilized phage primers.

### 2.9 Statistical analysis

Linear mixed-effects models (LMMs) were used to analyze both RT-qPCR and growth-curve data. For RT-qPCR, ΔCt values were modeled with condition as a fixed effect and biological replicate as a random effect. Pairwise comparisons among conditions were obtained from estimated marginal means, with *p*-values adjusted using the Benjamini–Hochberg false discovery rate (FDR) correction.

For bacterial growth, optical density at 600 nm was analyzed using LMMs with condition and time as fixed effects, biological replicate as a random effect, and time modeled using natural cubic splines (df = 4) to account for non-linear growth dynamics. Pairwise comparisons among conditions were computed from estimated marginal means at each observed time point, with Benjamini–Hochberg correction applied to *post hoc p*-values.

In both analyses, model significance was first assessed by ANOVA on the fitted LMMs, whereas multiple-testing correction was applied only to pairwise comparisons. Statistical significance was defined as *p* < 0.05.

Statistical analyses were performed in R using RStudio.^10^

## 3 Results

### 3.1 Phage host range

In this study, six isolated Trentingrana NWS phages were tested against 67 *L. helveticus* strains isolated from Trentingrana NWS (Table 1). The phages showed different host specificities, with the ability to induce lysis in other strains than their specific host. In particular, ϕCV244 and ϕP185 were able to infect more than one strain of *L. helveticus*, while ϕCR191, ϕS16, and ϕS193 induced lysis only in one strain besides their host.

### 3.2 Phage morphology

**Figure 1:**
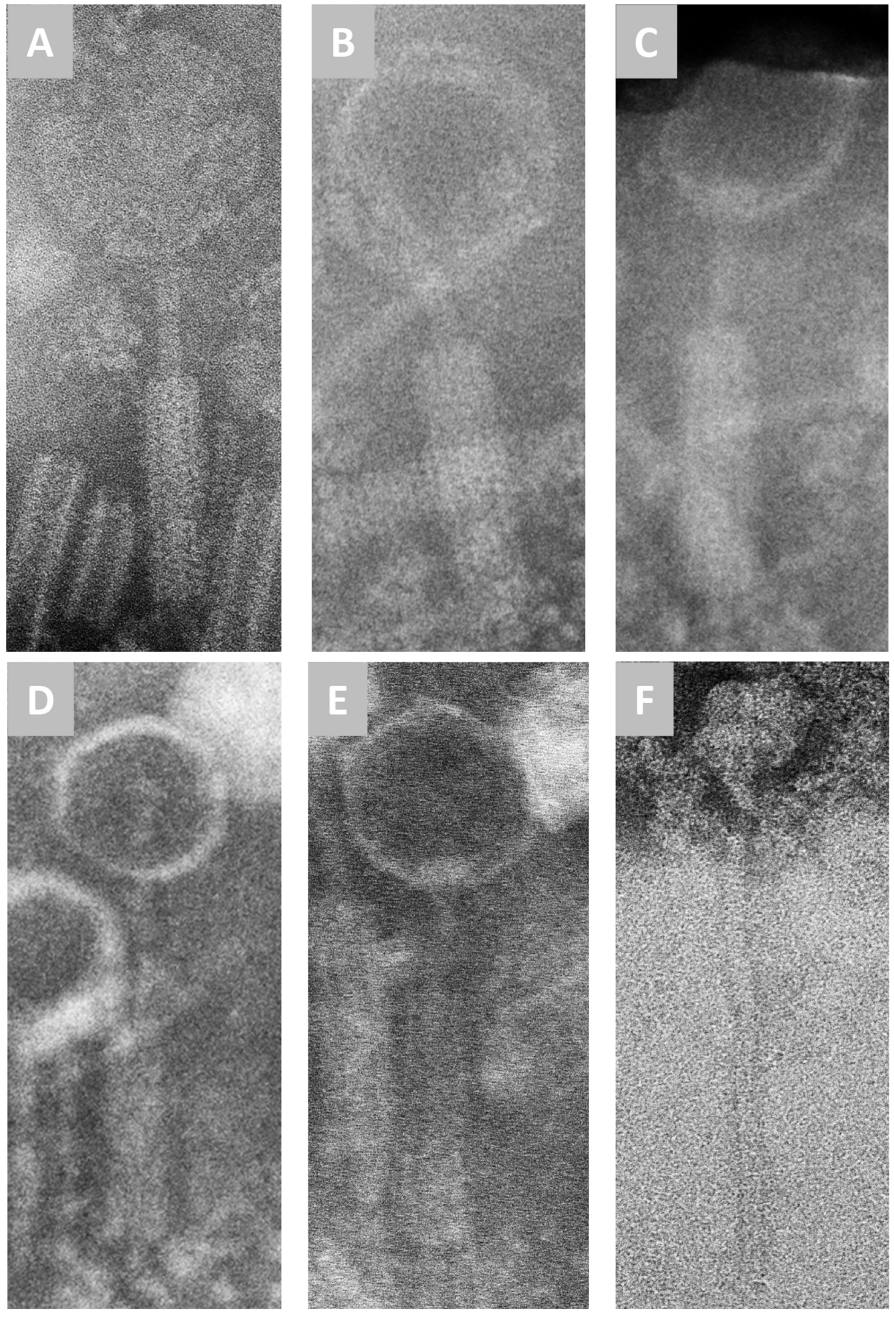
Transmission electron microscopy of the considered NWS phages. **(A)** ϕ CR191, **(B)** ϕ P185, **(C)** ϕ S16, **(D)** ϕ S193, **(E)** ϕT280, **(F)** ϕ CV244.

Electron microscopy was used to acquire information on the studied phages, based on their morphology. TEM images acquired at 120 k of magnification were suitable for virion measurements for all six phages. Characterized by an icosahedral head with both long contractile and non-contractile tails, these phages have been assigned to the class *Caudoviricetes* and displayed either myovirus-like (ϕCR191, ϕP185, ϕS16, ϕS193, ϕT180) or siphovirus-like morphotypes (ϕCV244). No clear tail fibers were observed; capsid and tail sizes for each phage are listed in Table 2, and phage images are shown in Figure 1.

### 3.3 Phage genomic characterization and comparison

General information about phage genomes is reported in Table 2. Genomic sequence information classified these phages as part of the *Caudoviricetes* class with an average genome size of 36.341 Kbp and an overall average G + C content of 37.10%. For each of our phages, the first hit of genome similarity through BLASTn corresponds to *L. helveticus* phage ϕAQ113 (NC_019782.1), with a similar genome size (36.566 Kbp) and G + C content (37%).

Genome annotation is presented in Supplementary Figure S1, with the summary of the obtained annotation data listed in Supplementary Table S2. Phage genomes contained on average ~72 putative ORFs, mainly divided based on the protein function into: virus structure (~14 ORFs), DNA packaging (~2 ORFs), host lysis (~3 ORFs), lysogeny and integration (~4 ORFs), DNA repair and replication (~4 ORFs), hypothetical protein (~42 ORFs).

One superinfection exclusion-associated gene was found in phages ϕS16 (ORF 50) and ϕS193 (ORF 58), while remaining phages harbored metallo-protease genes with potentially the same function (ϕCR191, ORF 24; ϕCV244, ORF 24; ϕP185, ORF 7; ϕT280, ORF 7). Putative integrase genes were found in five genomes, specifically in ϕCR191 (ORF 22), ϕCV244 (ORF 20), ϕP185 (ORF 10), ϕS16 (ORF 48) and ϕS193 (ORF 57). Other genes, potentially involved in phage genome integration/prophage lifestyle, were also found in phage ϕCR191 (ORF 25, ORF 28, ORF 29, ORF 44), ϕCV244 (ORF 26, ORF 28, ORF 31, ORF 33, ORF 34, ORF 54), ϕP185 (ORF 56, ORF 80), ϕS16 (ORF 59, ORF 60, ORF 61), ϕS193 (ORF 21) and ϕT280 (ORF 2, ORF 3, ORF 56). No t-RNA or auxiliary metabolic genes (AMGs) were detected.

Most of the annotated ORFs encode for hypothetical/unknown proteins present singularly or in multiple copies between genomes. In particular, we counted 39 ORFs for ϕCR191, 48 ORFs for ϕCV244, 49 ORFs for ϕP185, 47 ORFs for ϕS16, 31 ORFs for ϕS193 and 42 ORFs for ϕT280.

**Figure 2:**
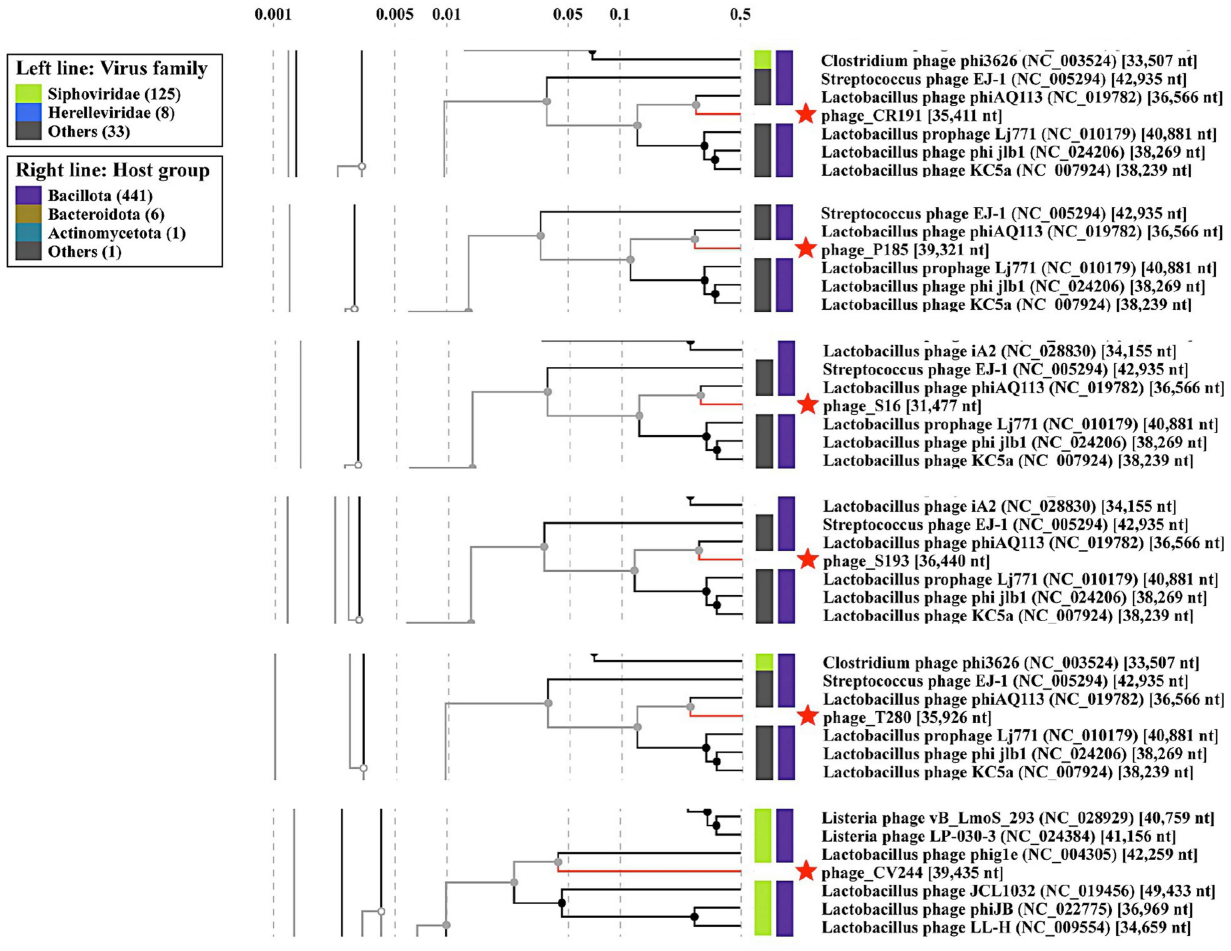
Proteomic trees were computed with ViPTree, respectively, between the considered phages and the relative proteomic database. The tree was constructed based on genome-wide tBLASTx similarities against the ViPTree reference database. Red stars indicate the prophages characterized in this study. Branch lengths reflect normalized genomic similarity scores. Colored bars indicate viral family (left) and host taxonomy (right).

Each genome was further characterized using ViPTree webserver to compare their global protein content (Figure 2). Phage ϕCR191, ϕP185, ϕS16 and ϕS193 were clustered with phage ϕAQ113 in the myovirus-like morphotype group of the tree, while ϕCV244 was grouped with phage phig1e (NC_004305.1), as part of the siphovirus-like morphotype group. According to these results, high overall similarity at the DNA level was shown in Supplementary Figure S2A for the myovirus-like morphotype phages and Supplementary Figure S2B for ϕCV244 with respect to the closest relatives’ genomes. Structural modules associated with head formation and DNA packaging (including terminase subunits) and tail morphogenesis (including tape measure proteins) were well conserved, whereas regions involved in regulation, integration, and host interaction showed higher variability.

Despite being classified within the class *Caudoviricetes*, the prophages analyzed in this study were assigned as singletons by vConTACT2, indicating limited shared protein content with reference phages under the applied parameters. In contrast, phage ϕAQ113, although also unclassified, showed sufficient similarity to be integrated into the gene-sharing network (Supplementary Figure S3). These results are consistent with a lower degree of shared gene content between the analyzed prophages and reference phages at the network level.

#### 3.3.1 Diversification of regulatory modules among Trentingrana prophages

**Figure 3:**
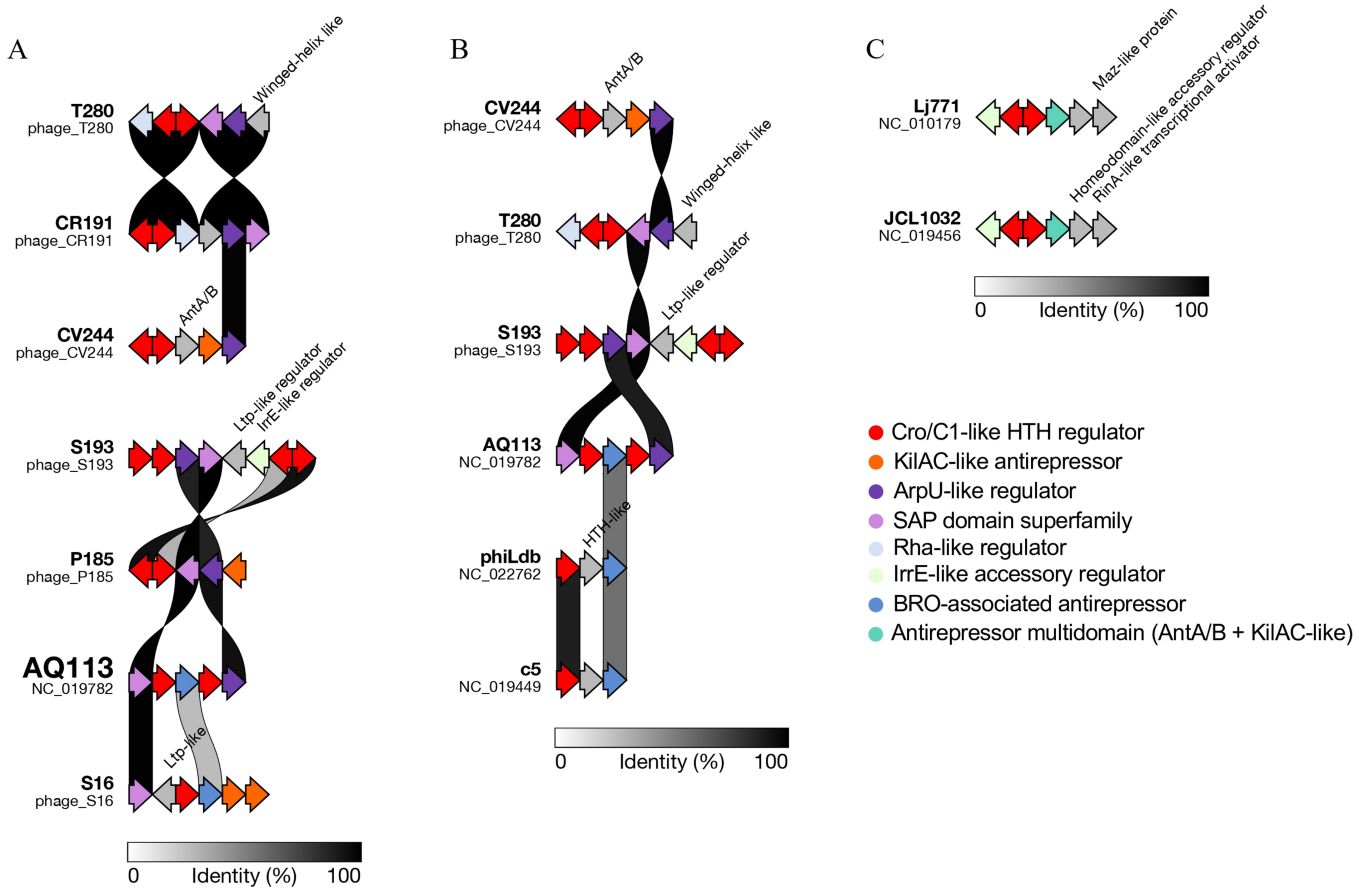
Comparative organization of regulatory modules in Trentingrana prophages and related dairy *Lactobacillus* phages. **(A)** Overview of putative lysogeny-control regions identified in the six Trentingrana prophages and reference phage ϕAQ113. **(B)** Comparison with representative myovirus-like dairy phages (ϕAQ113, phiLdb, and c5). **(C)** Comparison with siphovirus-like reference phages (Lj771 and JCL1032). Genes encoding transcriptional regulators and associated proteins, including Cro/C1-like repressors, antirepressors (e.g., KilAC-like), and accessory DNA-binding proteins (e.g., ArpU-, Rha-, BRO-, and IrrE-like), are highlighted. Homologous regions are connected based on sequence similarity (shaded areas; identity scale indicated). Arrows represent predicted coding sequences and their transcriptional orientation. For visualization purposes, regulatory regions are aligned according to sequence similarity and displayed in proximity, but may not be collinear or occupy equivalent genomic positions in the native genomes.

To investigate whether closely related Trentingrana prophages share conserved regulatory architectures or exhibit functional diversification, we compared the putative lysogeny-control regions of the six identified prophages with (i) those phage clustered nearby in the ViPTree analysis and, (ii) with selected representative dairy-associated *Lactobacillus* phages (Ldl1, NC_026609; c5, NC_019449; phiLdb, NC_022762; LL-H, NC_009554; LL-Ku, NC_022989), chosen based on high genome similarity and their relevance to dairy ecosystems (primarily infecting *Lactobacillus delbrueckii*), given the limited availability of publicly characterized *L. helveticus* phage genomes. By integrating InterProScan-based functional annotation, we aimed to evaluate both the degree of conservation of canonical regulators and the presence of divergent or unique regulatory elements, which could reflect distinct strategies of signal sensing, integration, and induction of the lytic cycle. Comparative visualization of the regulatory loci was shown in Figure 3. Although these regions are displayed in proximity for clarity, they represent functionally related modules that are not necessarily collinear in their native genomic context. In contrast to the relatively conserved structural and DNA-packaging backbone, the loci displayed substantial variability in both gene content and organization. In the overview comparison, ϕT280 and ϕCR191 shared a similar regulatory configuration centered on Cro/C1-like repressors and accessory DNA-binding proteins, including Rha-like and winged-helix-like regulators. In contrast, ϕS193, ϕP185, and ϕS16 displayed more diverse and rearranged combinations of Cro/C1-like, ArpU-like, KilAC-like, BRO-associated, Ltp-like, and IrrE-like regulators. Notably, ϕCV244 showed a more compact architecture, characterized by Cro/C1-like regulators associated with a multidomain antirepressor architecture including AntA/B- and KilAC-like elements. The reference phage ϕAQ113 showed an intermediate configuration, sharing part of the Cro/C1-centered backbone while differing in the composition of its accessory regulatory repertoire (Figure 3A). These trends became clearer in focused comparisons with representative external phages. In the comparison including ϕCV244, ϕT280, ϕS193, ϕAQ113, phiLdb, and c5, ϕAQ113 retained a relatively compact Cro/C1-centered organization, whereas phiLdb and c5 displayed reduced modules dominated by Cro/C1-like and HTH-associated functions. In contrast, our prophages showed more expanded and rearranged combinations of accessory regulators, including AntA/B-like, ArpU-like, Ltp-like, and IrrE-like proteins, indicating a continuum of regulatory-module complexity across related dairy phages rather than conservation of a single canonical cassette (Figure 3B). A further comparison with Lj771 and JCL1032 highlighted an additional regulatory configuration, in which Cro/C1-like regulators are associated with distinct accessory proteins, including Maz-like, homeodomain-like, and RinA-like transcription-related functions, further supporting the broad diversification of lysogeny-control modules among dairy-associated phages (Figure 3C).

**Figure 4:**
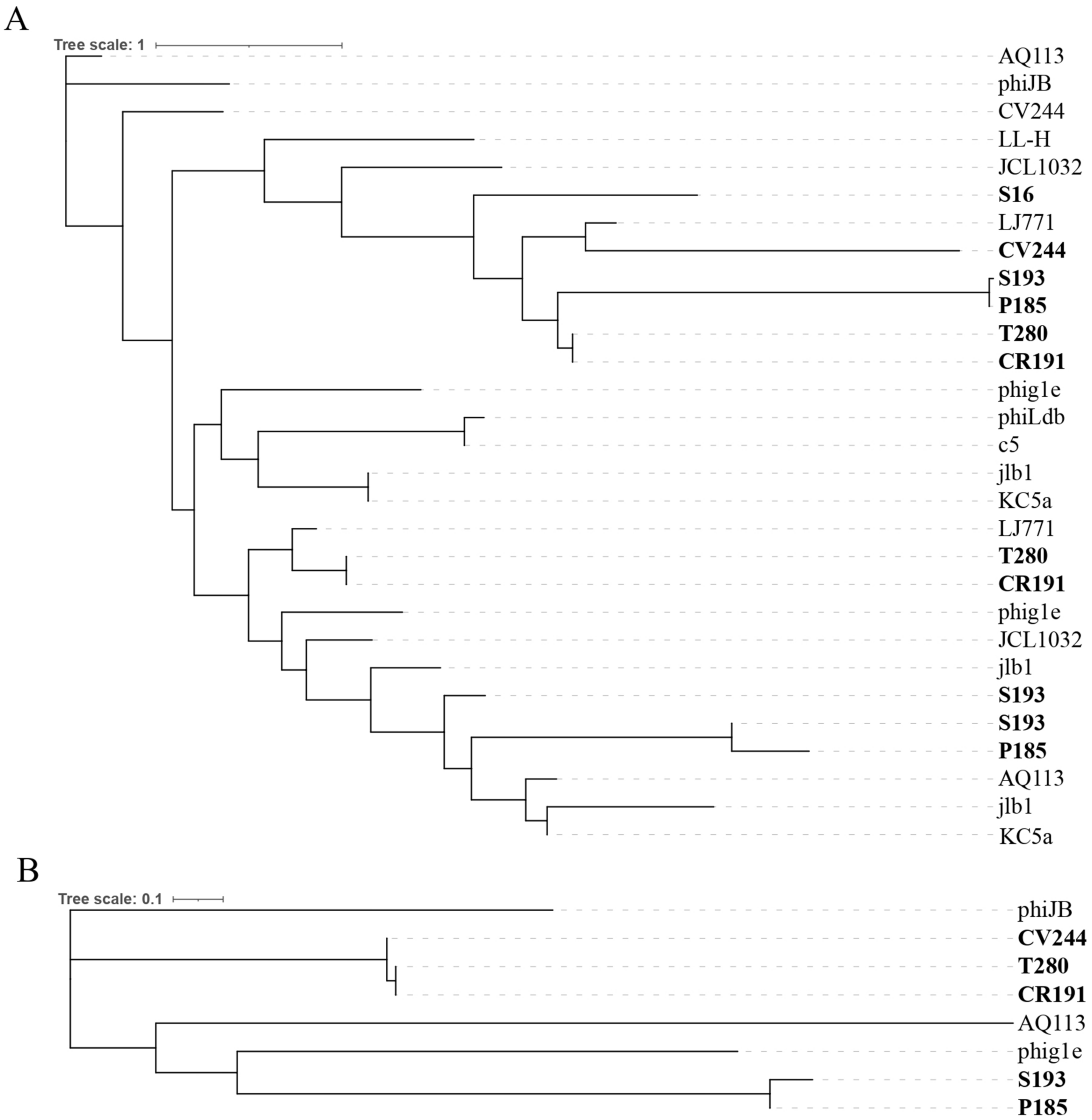
Phylogenetic relationships of selected regulatory proteins in the considered Trentingrana prophages and related dairy *Lactobacillus* phages: **(A)** Maximum-likelihood phylogeny of Cro/C1-like repressors and **(B)** Maximum-likelihood phylogeny of ArpU-like regulatory proteins. Protein sequences from Trentingrana prophages were aligned together with homologs from representative dairy-associated *Lactobacillus* phages, including ϕAQ113-, phiJB-, LL-H-, JCL1032-, Lj771-, phig1e-, phiLdb-, c5-, KC5a-, and jbl1- related genomes. Trees were inferred from MAFFT alignments using IQ-TREE under the best-fit substitution model and visualized in iTOL. Branch support values are indicated as bootstrap percentages. Prophages from this study are highlighted in bold.

Proteome-based phylogenetic analysis of regulatory proteins corroborated these observations. Cro/C1-like repressors (Figure 4A) and ArpU-like proteins (Figure 4B) did not cluster strictly according to the global proteome similarity relationships inferred by ViPTree, but instead showed interspersed affiliations with homologs from diverse dairy phages. Similar patterns were also observed for KilAC-like antirepressor, BRO-like associated antirepressor, IrrE-like accessory regulator, and Rha-like regulator (Supplementary Figures S4A–D), reinforcing the view that regulatory modules represent a major axis of diversification among otherwise related prophages.

The full set of aligned regulatory proteins identified across Trentingrana and reference phages, including domain assignments and sequence clustering, is reported in Supplementary Table S3.

### 3.4 Investigation of prophage DNA replication

Prophage DNA replication dynamics were investigated using a three-part experimental approach under defined stress conditions, including (i) quantification of changes in phage DNA copy number, (ii) monitoring of bacterial growth, and (iii) detection of extracellular phage particles in the supernatant. Sampling time points were selected to capture early and intermediate stages of phage activation. In particular, the 2 h time point was used to assess early changes in phage DNA copy number, whereas the 4 h time point was chosen to allow progression toward potential virion assembly and release. However, these intervals did not capture the full temporal dynamics of induction and should therefore be interpreted as a targeted snapshot of early phage dynamics rather than a complete kinetic characterization.

**Figure 5:**
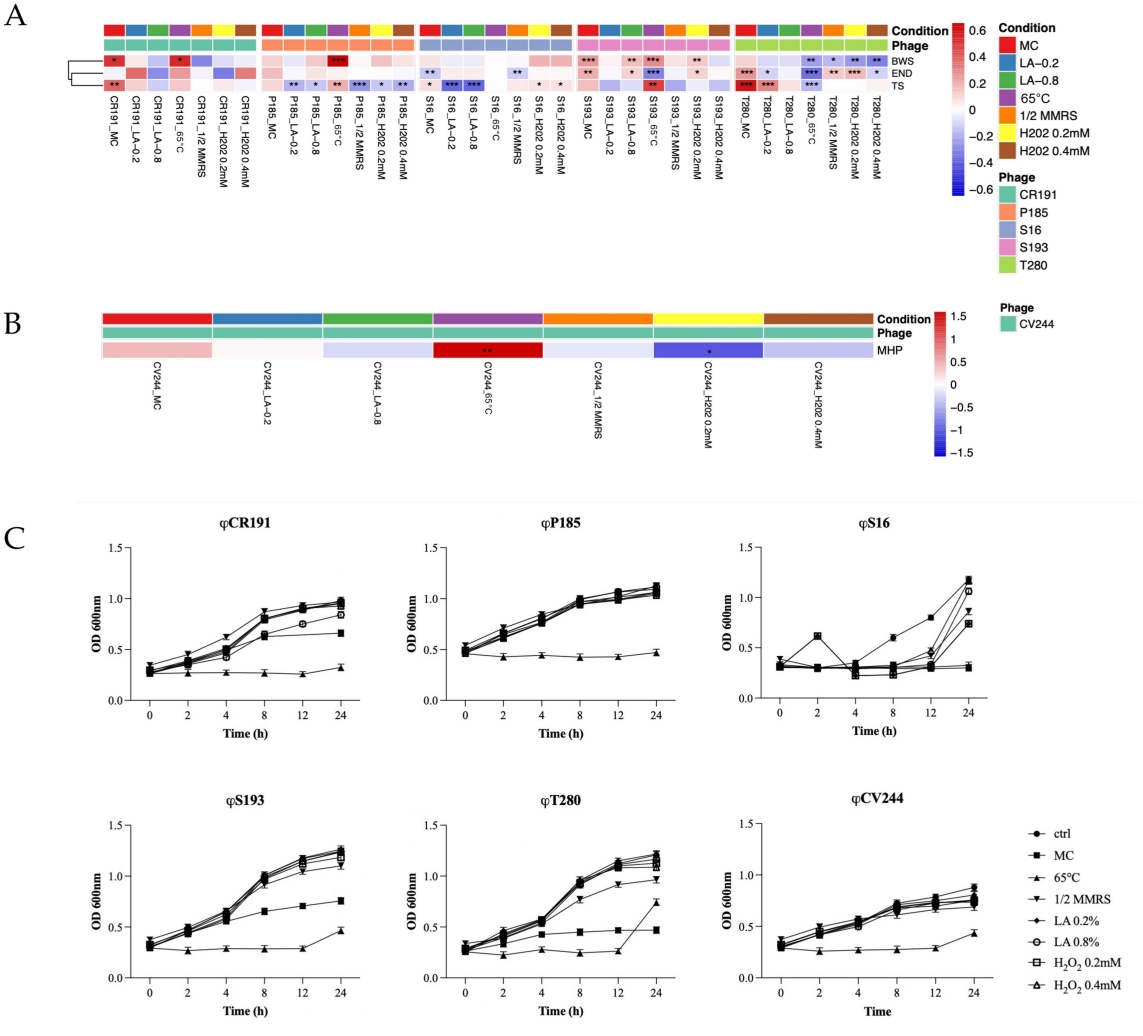
Heatmap showing prophage induction levels after 2 h of exposure to different stress conditions for phages ϕCR191, ϕP185, ϕS16, ϕS193, and ϕT280 **(A)**, and ϕCV244 **(B)**. Data are expressed as log_2_ fold changes calculated using the ΔΔCt method. Asterisks indicate statistically significant differences compared to control conditions. **(C)** Representative growth curves (OD600) of the corresponding *Lactobacillus helveticus* strains under the tested conditions. Data are presented as mean ± SD, *N* = 3. For linear mixed-effects model results, see Supplementary Tables S4 and S5.

Heatmaps in Figures 5A,B show changes in phage DNA copy number across selected gene sets after 2 h of exposure to seven stressor conditions. For phage ϕCR191, ϕP185, ϕS16, ϕS193, and ϕT280, prophage-specific genes representing distinct functional modules were selected, including adsorption/host-recognition, baseplate wedge subunit (BWS); tail morphogenesis, tail sheath (TS); lysis, endolysin (END), in order to minimize false negatives due to partial induction or local genome rearrangements. In the case of ϕCV244, a single, prophage-specific, single-copy structural gene (minor head protein, MHP) was selected as the most reliable RT-PCR marker due to the lack of conserved suitable regions in other modules. The log_2_FC values were calculated on total DNA relative to the housekeeping gene *tuf* and therefore reflected changes in phage genomic copy number rather than transcriptional activity.

Overall, stress-induced changes in phage DNA copy number were modest in magnitude but statistically supported by linear mixed-effects models. For most myovirus-like morphotype prophages, responses remained within approximately ±0.6 log_2_FC, whereas ϕCV244 displayed a broader range, reaching up to about +1.5 under heat stress and marked negative values under oxidative stress (Figures 5A,B). LMM-based ANOVA indicated a significant overall effect of treatment for all markers in ϕCR191, for the single marker of ϕCV244, and for all three markers in ϕP185. In ϕS16, significant effects were detected for tail sheath and endolysin but not baseplate wedge subunit, whereas all markers were significantly affected in both ϕS193 and ϕT280. Please refer to Supplementary Tables S4 and S5 for full model outputs, including ANOVA and pairwise comparisons.

When only contrasts with the control were considered, the strongest and most recurrent responses were observed under MC and heat stress, although the overall pattern remained strongly dependent on both the prophage and the selected marker/gene. In ϕCR191, MC treatment was associated with significant increases in BWS and TS markers (log_2_FC ~ +0.5, *p* < 0.05; log_2_FC ~ +0.4, *p* < 0.01, respectively), while END did not show significant changes in any condition. In ϕCV244, heat shock produced the strongest increase in DNA copy number (log_2_FC ~ +1.5, *p* < 0.01), whereas oxidative stress (H_2_O_2_ 0.2 mM) led to a significant decrease of the MHP marker (log_2_FC ~ −1, *p* < 0.05). In ϕP185, the most consistent responses involved the TS marker, which varied significantly across multiple conditions, including heat (log_2_FC ~ +0.2, *p* < 0.01), lactic acid (log_2_FC ~ −0.2, *p* < 0.01 at 0.2%; log_2_FC ~ −0.1, *p* < 0.05 at 0.8%), oxidative stress (log_2_FC ~ −0.1, *p* < 0.05 at 0.2 mM; log_2_FC ~ −0.2, *p* < 0.01 at 0.4 mM), and medium dilution (log_2_FC ~ −0.3, *p* < 0.001), while BWS responded specifically to heat (65 °C, log_2_FC ~ +0.6, *p* < 0.001) whereas END showed no significant control-based contrasts. In ϕS16, TS again represented the most responsive marker, with significant variation under MC (log_2_FC ~ +0.2, *p* < 0.05), both lactic acid concentrations (log_2_FC ~ −0.5, *p* < 0.001) and both H_2_O_2_ concentrations (log_2_FC ~ +0.1, *p* < 0.05), whereas END differed significantly only under MC (log_2_FC ~ −0.1, *p* < 0.01) and medium dilution (log_2_FC ~ −0.1, *p* < 0.01). In ϕS193, both BWS and END exhibited significant changes across several conditions: MC (log_2_FC ~ +0.2, *p* < 0.01 BWS; log_2_FC ~ +0.3, *p* < 0.001 END), 0.8% lactic acid (log_2_FC ~ +0.1, *p* < 0.05 BWS; log_2_FC ~ +0.2, *p* < 0.01 END), heat (log_2_FC ~ −0.5, *p* < 0.001 BWS; log_2_FC ~ +0.4, *p* < 0.001 END) and H_2_O_2_ 0.2 mM (log_2_FC ~ +0.1, *p* < 0.05 BWS; log_2_FC ~ +0.2, *p* < 0.01 END). TS showed a more restricted response (heat: log_2_FC ~ −0.5, *p* < 0.01). Finally, in ϕT280, all markers showed significant ctrl-based contrasts, but with distinct patterns: BWS was mainly affected by heat (log_2_FC ~ −0.3, *p* < 0.01), oxidative stress (log_2_FC ~ −0.3, *p* < 0.01 at 0.2 mM; log_2_FC ~ −0.5, *p* < 0.01 at 0.4 mM), and nutrient limitation (log_2_FC ~ −0.1, *p* < 0.05). TS responded primarily to MC (log_2_FC ~ +0.6, *p* < 0.001), 0.2% lactic acid (log_2_FC ~ +0.4, *p* < 0.001) and heat (log_2_FC ~ −0.2, *p* < 0.001); END showed the broadest response across conditions (MC: log_2_FC ~ +0.4, *p* < 0.001; 0.2% lactic acid: log_2_FC ~ −0.1, *p* < 0.05; heat: log_2_FC ~ −0.5, *p* < 0.001; medium dilution: log_2_FC ~ +0.2, *p* < 0.01; 0.2 mM H_2_O_2_: log_2_FC ~ +0.2, *p* < 0.001; 0.4 mM H_2_O_2_: log_2_FC ~ −0.1, *p* < 0.05). Given that RT-qPCR quantifies phage DNA rather than transcriptional activity, the observed patterns likely reflect early or partial stages of induction; confirming transcriptional activation and distinguishing partial versus productive cycles will require future RNA-seq, multi-time-point, or single-cell approaches.

In parallel, bacterial growth was monitored by optical density over 24 h under the same stress conditions, and the resulting trajectories were compared with the untreated controls. As shown in Figure 5C, growth responses were strongly time-dependent and differed among strains. Spline-based linear mixed-effects models confirmed a highly significant effect of time and, importantly, a highly significant condition-by-time interaction in all strains, whereas the overall main effect of condition alone was not significant. This indicates that stress treatments did not simply reduce growth uniformly, but instead altered the shape and timing of the growth curves in a strain-specific manner.

Overall, heat shock at 65 °C produced the strongest and most persistent growth inhibition, while Mitomycin C also caused marked suppression in selected strains, most clearly in *Lactobacillus helveticus* T280. By contrast, lactic acid, oxidative stress, and nutrient limitation generally produced more limited or strain-dependent effects, often allowing partial or complete recovery at later time points. Consistent with the trajectories shown in Figure 5C, strain S16 displayed the most heterogeneous kinetics, with several treatments remaining close to baseline during the early phase and then diverging sharply after 8–12 h, whereas CV244 showed the overall slowest growth profile across conditions. Pairwise comparisons by time further indicated that significant differences were usually absent or modest at the beginning of the experiment and became progressively more evident at later time points, particularly under heat stress and, in some strains, under MC (see Supplementary Table S4 and S5 for full model outputs, including ANOVA and pairwise comparisons).

Supernatant phage detection after 4 h (Table 3) showed, as expected, a strong inducing effect of MC across all the tested prophages. A similar pattern was observed after heat shock exposure, while the remaining stressors produced more selective prophage DNA replication. Particularly, phage ϕP185 and ϕS193 were induced by all the conditions, while ϕCV244 was sensitive only to MC and 65 °C. Apart from MC treatment, only lactic acid induced prophages ϕS16, while ϕT280 was not induced under oxidative stress. ϕCR191, ϕCV244, and ϕS16 were not detected under nutrient limitation conditions.

**Table 3:** Supernatant phage detection across the tested conditions after 4 h.

Phage	Condition							
	Ctrl	MC	65 °C	1/2 MMRS	LA 0.2%	LA 0.8%	H_2_O_2_ 0.2 mM	H_2_O_2_ 0.4 mM
CR191	NA	1.29 ± 0.18	1.4 ± 0.18	NA	0.48 ± 0	0.37 ± 0.18	NA	0.52 ± 0.18
CV244	NA	1.08 ± 0.3	0.9 ± 0	NA	NA	NA	NA	NA
P185	NA	1.26 ± 0.3	1 ± 0.3	0.37 ± 0.18	NA	0.95 ± 0	NA	0.85 ± 0
S16	NA	1.18 ± 0.3	NA	NA	0.67 ± 0.18	0.82 ± 0.18	NA	NA
S193	NA	1.23 ± 0.3	0.78 ± 0	0.88 ± 0.18	NA	0.85 ± 0	0.75 ± 0.18	NA
T280	NA	1.04 ± 0	0.88 ± 0.18	0.8 ± 0.18	0.87 ± 0.18	NA	NA	NA

Data were expressed as means ± standard deviation of the phage titer as pfu/ml, *N* = 3. Ctrl, control; MC, mytomicin C; LA, lactic acid.

## 4 Discussion

In Trentingrana cheese production, cheesemaking performance and final distinctive organoleptic characteristics rely on the activity of natural whey starters (NWS). Typically, the presence of bacteriophages does not disrupt the technological performance of the NWS. However, in specific conditions, such as the introduction of new phages and/or decreased phage tolerance in certain lactobacilli, phage-mediated decline in NWS fitness may be observed (White et al., 2022).

In this study, we characterized six prophages isolated from *Lactobacillus helveticus* strains originating from different Trentingrana NWS ecological consortia. The host range of these isolated phages was limited and largely restricted to the host strains from which the phages were originally isolated, confirming previous observations for other dairy-associated phages (Weinbauer, 2004; Paillet et al., 2022). This high specificity supports a close co-evolutionary relationship between phage and its bacterial host and, from a technological perspective, may contribute to maintaining bacterial biodiversity within the NWS. Although host range was assessed exclusively within *Lactobacillus helveticus*, the strain panel was selected to include isolates originating from different dairy plants and NWS systems. These strains likely represent distinct microbial ecosystems and a diverse repertoire of genomic and proteomic traits, including variability in cell surface structures, receptor availability, and phage defense systems. Nevertheless, potential interactions with other dominant members of NWS communities, such as *Lactobacillus delbrueckii* subspecies, cannot be excluded, and future host-range analyses in multi-species systems will be important to better capture the ecological complexity of natural whey starter ecosystems.

Our phages showed high sequence homology with the available genome of phage ϕAQ113 (NC_019782.1), belonging to the same ecosystem (Grana cheeses NWS) (Zago et al., 2013), supporting their placement within the class *Caudoviricetes*. In contrast, ϕCV244 showed limited similarity to the myovirus-like morphotype on BLASTn, and by TEM microscopy was recognized as belonging to the siphovirus-like morphotype (Casjens, 2011; Bebeacua et al., 2013; Feyereisen et al., 2019; Guo et al., 2022). Notably, no canonical tail fiber proteins were identified in any of the phage genomes, suggesting that host recognition may be mediated by proteins currently annotated as hypothetical or of unknown function. Across the myovirus-like morphotype phage group, the genome comparisons highlighted a conserved functional architecture, including integrases, transcriptional regulators, anti-repressors, and recombination-associated proteins such as Sak/Sak4-like annealing proteins, HNH endonucleases, and Holliday junction resolvases. These features are consistent with a temperate lifestyle and the capacity for stress-induced activation (Brady et al., 2021). Importantly, differences between closely related prophages were primarily localized within regulatory and accessory regions rather than structural modules, suggesting that variation in lysogeny-control systems may drive functional diversification and underlie the heterogeneous induction phenotypes observed in our experiments. Consistently, in ϕCV244, the genome retained the same functional “grammar” (integration-excision, lysis, structural morphogenesis, and DNA metabolism), with clear conservation of major morphogenesis genes against the reference (*Lactobacillus* phage phig1e, NC_004305.1) and a structured regulatory region enriched in transcriptional regulators and anti-repressors. Notably, differences between ϕCV244 and the reference concentrated in the regulatory and accessory segments rather than in the morphogenetic backbone, consistent with the general observation that phage genomes are constrained by essential replication-assembly requirements, while regulatory and host-interaction functions diversify more rapidly (Grigson et al., 2025).

To interpret the mechanistic basis of the heterogeneous induction patterns observed in this study, we examined the organization and evolutionary relationships of prophage regulatory modules. Comparative analyses showed that genomic variability is primarily concentrated within lysogeny-control regions, while the broader genomic backbone remains comparatively conserved. This pattern is consistent with the modular and mosaic organization of temperate-phage genomes, in which substantial diversity can persist despite conservation of core genome architecture (López-Leal et al., 2022). Accordingly, our six Trentingrana prophages encode distinct combinations of Cro/C1-like repressors, anti-repressors, and accessory DNA-binding proteins, including ArpU-, Rha-, KilAC-, BRO-, and IrrE-like regulators. Recent studies indicate that relatively small regulatory cassettes can substantially alter prophage fate decisions, even among closely related or co-resident prophages (Brady et al., 2021; Silpe et al., 2023a). Consistent with this, our data suggest that diversification of lysogeny-control modules provides a flexible genetic basis for tuning prophage responsiveness to environmental cues. The distribution of functionally analogous but non-identical regulatory repertoires across related dairy-associated phages (phiLdb, c5, Lj771, and JCL1032) further supports this interpretation, indicating that regulatory-module composition, rather than virion-building capacity *per se*, appears to be an important determinant of prophage behavior.

The compact regulatory architecture of ϕCV244, together with its distinct proteome-based phylogenomic placement, may contribute to its differentiated response to stress. In contrast, ϕAQ113-related prophages share a conserved genomic backbone but differ in regulatory composition, suggesting that fine-scale rewiring of lysogeny-control modules may modulate sensitivity to environmental cues and enable intermediate activation states. Phylogenetic analyses support this view: regulatory proteins (Cro/C1-like, ArpU-like, KilAC-, BRO-, IrrE-, and Rha-like) do not cluster according to whole-proteome relationships, but instead show interspersed affiliations with homologs from diverse dairy phages. This decoupling between proteome similarity and regulatory composition suggests recurrent recombination and modular exchange of lysogeny-control elements, identifying regulatory modules as a major axis of diversification in dairy-associated prophages. This interpretation is further supported by recent experimental evidence showing that prophage regulation can involve accessory modules beyond the canonical repressor-centered switch, including systems integrating prophage control with broader host physiological states (Silpe et al., 2023b; Guo et al., 2024).

In NWSs, prophages with conserved morphogenesis backbones but variable regulatory and accessory repertoires may differ in inducibility under process-related stresses (Mancini et al., 2021; Wu et al., 2023; Neviani et al., 2024; Lin et al., 2026). The responses to different stressors revealed distinct and condition-specific induction patterns, highlighting that prophage activation is not governed by a single universal trigger but rather by stress-dependent mechanisms. This interpretation is supported by the LMM-based analysis performed, which showed that stress effects are not uniform but strongly prophage- and gene-set dependent, with significant deviations from control restricted to specific gene modules and conditions rather than a coordinated genome-wide response. Within this framework, back-slopping in Grana-like cheese production may be affected by biotic and abiotic fluctuations. In these terms, heat shock during the curd cooking step, increased lactic acid levels, oxidative stress, and depletion of nutrients in the NWS fermenter may affect host/prophage homeostasis within NWS communities, thereby potentially triggering the prophage lytic cycle (Ho et al., 2016; Mayo et al., 2021; Louw et al., 2023; Neviani et al., 2024). The prophage induction assays further support this view. Stressors elicited prophage-specific responses, frequently characterized by a limited concordance between early increases in phage DNA copy number (2 h) and the subsequent detection of extracellular phage particles (4 h). This is consistent with the idea that prophage “induction” does not occur as an all-or-nothing process, but rather as a continuum ranging from partial excision/replication to complete virion production and host cell lysis (Henrot and Petit, 2022). Importantly, the LMM results indicate that MC and heat represent the most consistent drivers of changes in phage copy number relative to control, whereas other stressors produce more selective and condition-dependent responses. Heat stress induced the strongest increase in DNA copy number in ϕCV244 and significant responses in multiple markers in myovirus-like prophages, while oxidative stress frequently resulted in reduced or inconsistent DNA levels. Similarly, lactic acid and nutrient limitation produced variable effects, often restricted to specific modules such as tail or lysis genes, rather than coordinated induction across the genome. Because RT-qPCR quantifies phage DNA rather than transcriptional activation, these measurements likely capture early or intermediate stages of induction. In this context, the observed log_2_FC shifts, although modest, are consistent with an early or intermediate state that does not necessarily culminate in virion assembly and release. While MC consistently induced both DNA increase and particle release, other stressors often triggered detectable changes in DNA copy number without corresponding extracellular phage production, suggesting that progression beyond early induction stages is conditional and frequently constrained. This decoupling is particularly evident under oxidative stress and nutrient limitation, where modest or negative shifts in DNA abundance are likely to reflect impaired replication or incomplete induction. The 2 h heat stress provides a clear example of this phenomenon: despite inducing strong increases in phage DNA and subsequent particle detection for several prophages, it also caused marked growth inhibition (low/flat 600 nm OD), suggesting that host physiological constraints may delay or limit completion of the lytic cycle.

Across the 2 h lactic acid treatments, data showed a dose-related response, whereby stronger acidification could lead to negative log_2_FC values in selected phage DNA genes, particularly in ϕS16. Phage release at 4 h was consistent with this threshold-like behavior: extracellular particles were detected under intermediate lactic acid conditions (0.2–0.4%) for several prophages; however, detection was not consistently observed across all phages and appeared to be phage-dependent. Mechanistically, lactic acid stress alters the proton motive force, intracellular pH, and cellular redox balance, generating signals that can weaken lysogenic repression or activate alternative stress-response pathways without necessarily triggering a canonical SOS response (Brady et al., 2021). This interpretation is supported by the real-time PCR data, including those obtained for phage ϕCV244 (Table 3), and it is consistent with observations in food-relevant systems showing that abiotic stressors such as organic acids can modulate prophage-associated outcomes in a concentration-dependent manner (Oliveira et al., 2017; Lu and Guo, 2024). The oxidative stress conditions were particularly informative because they showed dose-dependent signals in most of the phages, where higher H_2_O_2_ concentrations were associated with a repression trend. At 4 h, particle detection was selective and did not always track the early DNA induction signals: some conditions showed particle detection only at the higher dose for certain phages, while other phages showed no detectable particles despite earlier RT-PCR changes. Sub-lethal levels of reactive oxygen species (ROS) could activate RecA and permissively promote prophage replication. However, higher ROS concentrations become bactericidal or inhibit DNA replication and translation, thereby preventing late gene expression and virion assembly even when early excision or genome replication has already occurred (Brady et al., 2021; Zhang et al., 2022). Consequently, the 2 h RT-PCR measurements likely captured an “attempted induction” state, whereas phage particle detection at 4 h reflected the successful completion of the virion production pipeline. Reduced nutrient availability (half-strength MMRS) resulted only in minor changes at 2 h and limited phage particle detection at 4 h, restricted to a subset of prophages. In applied contexts such as fermentation, nutrient limitation may increase the likelihood that an additional stressor pushes the system beyond the induction threshold, particularly for prophages that are highly responsive under moderate stress but collapse under harsher conditions (Henrot and Petit, 2022; Zhong et al., 2025). It has been shown that nutrient availability, particularly its temporal dynamics, plays a key role in shaping interactions between phage life-history traits, initial conditions, and population dynamics. Lysis of nutrient-consuming competitors can remove energy from the system, linking initial lysogen frequency to final population density. Although this effect was limited in simple competitions, such phage-nutrient interactions are likely to have greater importance in complex microbial communities, warranting further investigation (Thomas et al., 2024).

Within this framework, the heterogeneous and dose-dependent responses to abiotic stressors observed in Trentingrana NWS cultures likely reflect distinct but functionally analogous regulatory architectures. The strong early increase in phage DNA observed for ϕCV244 under heat stress is consistent with a regulatory configuration permissive to rapid derepression, whereas ϕAQ113-like prophages appear to span a spectrum of related but non-equivalent regulatory organizations, potentially enabling graded induction responses such as genome amplification without full virion production. Single-cell studies have shown that phage production and prophage-linked lysis can be highly heterogeneous across individual cells, indicating that bulk measurements may mask variation in the fraction of cells crossing the induction threshold under a given condition (Wu et al., 2021; Kannoly et al., 2023). Although functional validation will be required to assign specific roles to individual regulators, synteny and phylogenetic evidence suggest that diversification of lysogeny-control modules represents a plausible genetic basis for the variability in induction dynamics reported here.

These findings are consistent with emerging models in which prophage activation is not strictly binary but modulated by regulatory-module composition, enabling context-dependent responses to environmental stresses and intermediate states such as genome amplification without full virion production (Silpe et al., 2023a,b).

Overall, these findings are particularly relevant for traditional food fermentations, which rely on inherently variable, spontaneously derived microbiota. In this context, back-slopping stabilizes process performance while driving the long-term evolution of complex microbial ecosystems. Within these systems, temperate phages emerge as active ecological modulators rather than passive genetic elements: through stress-dependent and often incomplete induction, they may reshape host population structure, promote strain turnover, and release intracellular resources without causing community collapse. Over successive cycles, these effects are likely to contribute to the robustness and adaptability of NWS consortia, ultimately influencing fermentation stability and product consistency.

## 5 Conclusion

This study characterized six temperate bacteriophages isolated from natural whey starters (NWS), revealing typical genomic features alongside the presence of large, conserved nucleotide regions of still unknown function. The observed spectrum of outcomes, ranging from partial, non-productive induction to full virion release, supports a model in which prophages contribute to population-level resilience by enabling controlled strain turnover, metabolic redistribution, and adaptive flexibility during back-slopping cycles. These findings suggest that such “domesticated” phages, while not impacting the starter acidification performance, may play beneficial and previously underappreciated roles within the microbial community. Further investigation in controlled NWS-like experimental systems will be essential to clarify the ecological and functional impact of these prophages and their contribution to the stability and quality of Grana-like cheese production, including Trentingrana.

## Data Availability

The datasets presented in this study can be found in online repositories. The names of the repository/repositories and accession number(s) can be found below: https://www.ncbi.nlm.nih.gov/genbank/, OQ627803.1, OQ627804.1, OQ627805.1, OQ627806.1, OQ627807.1, OQ627808.1.

## Footnotes

1
https://nanoporetech.com/products/minion

2
https://nanoporetech.com/

3
https://github.com/rrwick/Filtlong

4
https://github.com/nanoporetech/medaka

5
https://github.com/deprekate/PHANOTATE

6
https://github.com/gbouras13/pharokka

7
https://usegalaxy.eu

8
https://www.viptree.jp/viptree

9
www.snapgene.com

10
www.r-project.org

## Supplementary material

SUPPLEMENTARY TABLE S1 List of utilized primers for phages genes real time PCR.

SUPPLEMENTARY TABLE S2 Gene presence and annotation.

SUPPLEMENTARY TABLE S3 set of aligned regulatory proteins identified across Trentingrana and reference phages.

SUPPLEMENTARY TABLE S4 Linear mixed-effect full model output.

SUPPLEMENTARY TABLE S5 Linear mixed-effect full model output.

SUPPLEMENTARY FIGURE S1 Genomic maps of the six considered phages shown in circular form using SnapGene software.

SUPPLEMENTARY FIGURE S2 Complete sequences alignment of considered phages using LoVis4U software.

SUPPLEMENTARY FIGURE S3 Gene-sharing network analysis using vContact2.

SUPPLEMENTARY FIGURE S4 Phylogenetic relationships of accessory regulatory proteins in Trentingrana prophages and related dairy *Lactobacillus* phages.
