# Supplementary material for: Genomic and functional analysis of stress-responsive prophages in Lactobacillus helveticus
Source: Front Microbiol. 2026 Apr 23;17:1819103. doi: 10.3389/fmicb.2026.1819103 (PMC13149235; doi:10.3389/fmicb.2026.1819103)
Supplement: SUPPLEMENTARY TABLE S1 — List of utilized primers for phages genes real time PCR. [file Table_1.docx]

**Table S1.** List of utilized primers for genes amplification.

| **Primer name** | **Primer sequence** | **Target gene** | **Source/reference** |
| --- | --- | --- | --- |
| **Tuf-F** | 3’- TTACAAGGCGACAAGGAAGC -5’ | tuf | Desfossés-Foucault E. et al. 2012  doi.org/10.3389/fmicb.2012.00350 |
| **Tuf-R** | 3’- CGACCTGAAGCAACAGTACC -5’ |  |  |
| **END-F** | 3’- GAAGAGCGAGGCAATTATCC -5’ | endolysin | this study |
| **END-R** | 3’- TGCCTAAACTGACATCAAGAAC -5’ |  |  |
| **TS-F** | 3’- TGCAAGATGAGTTTGGACGCA -5’ | tail sheath | this study |
| **TS-R** | 3’- ACCTTCTCGCATCTTCTGTGC -5’ |  |  |
| **BWS-F** | 3’- GCTCCTGCAGCTATGGTTAGT-5’ | baseplate wedge subunit | this study |
| **BWS-R** | 3’-TCTGCAAAAGTTGCCGCATA -5’ |  |  |
| **MHP-F** | 3’- GAAGAGCGAGGCAATTATCC -5’ | minor head protein | this study |
| **MHP-R** | 3’- TGCCTAAACTGACATCAAGAAC-5’ |  |  |
