# Supplementary figures and images for: Genomic and functional analysis of stress-responsive prophages in Lactobacillus helveticus
Source: Front Microbiol. 2026 Apr 23;17:1819103. doi: 10.3389/fmicb.2026.1819103 (PMC13149235; doi:10.3389/fmicb.2026.1819103)

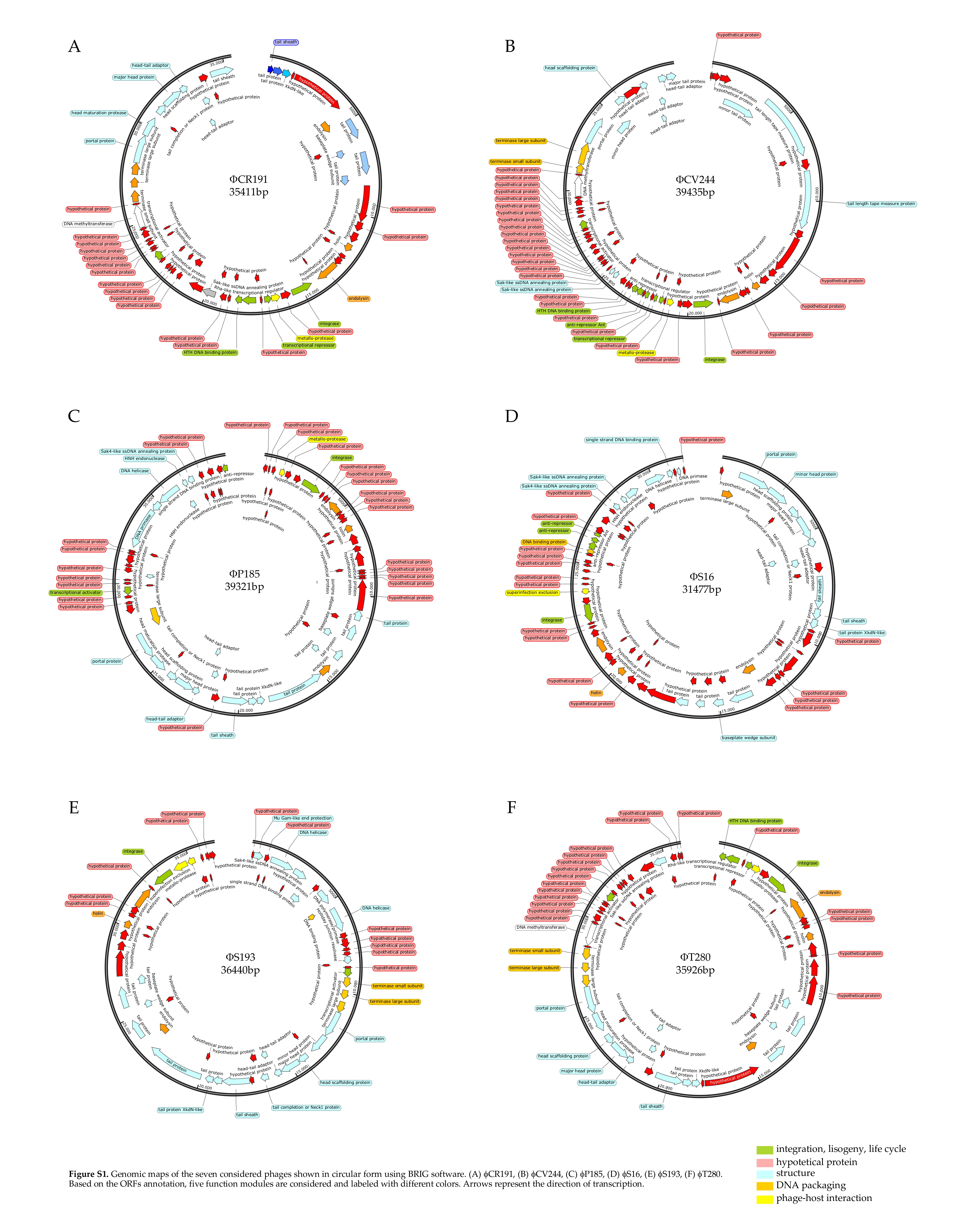

Supplement: SUPPLEMENTARY FIGURE S1 — Genomic maps of the six considered phages shown in circular form using SnapGene software. [file Image_1.JPEG]

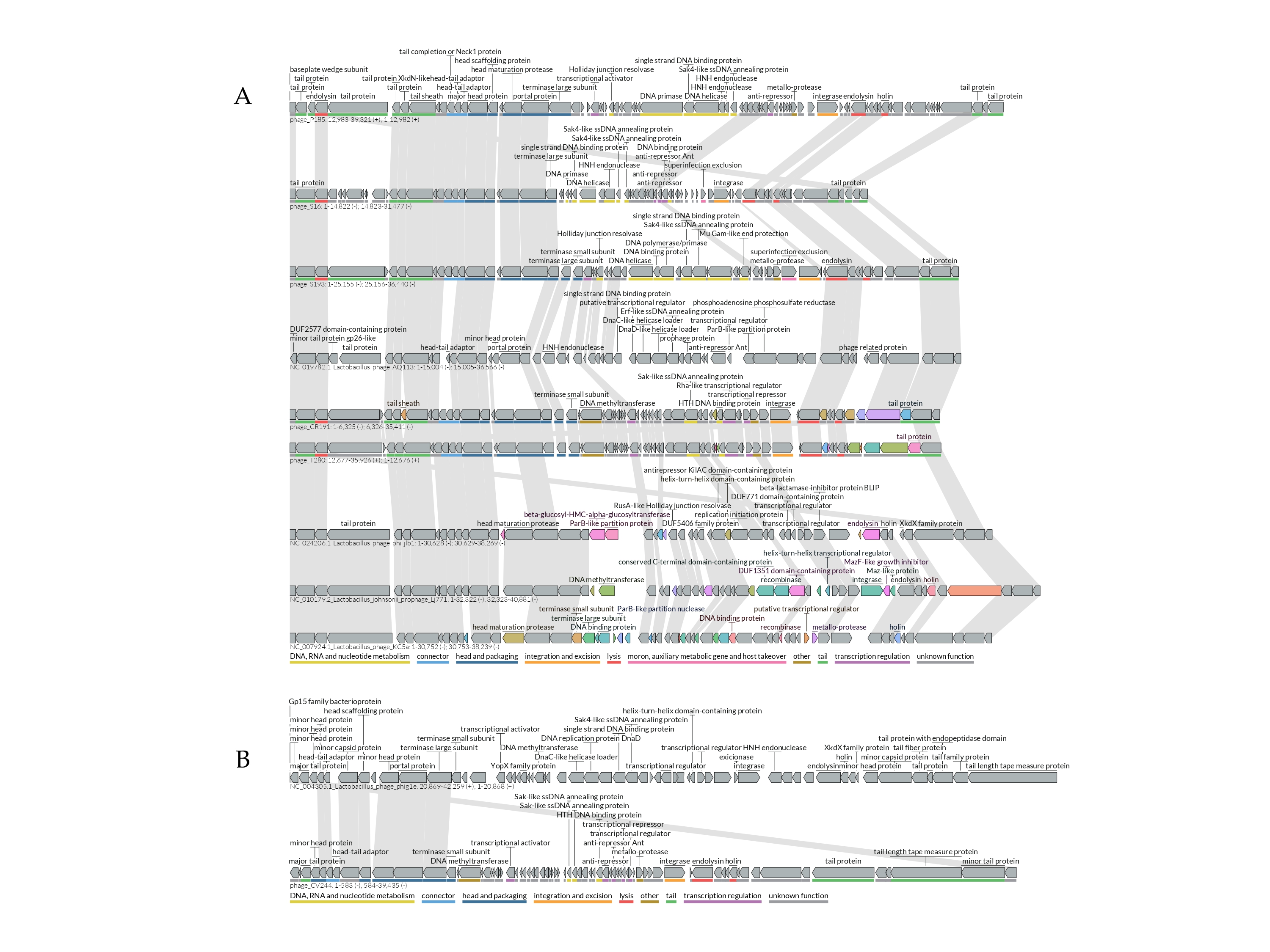

Supplement: SUPPLEMENTARY FIGURE S2 — Complete sequences alignment of considered phages using LoVis4U software. [file Image_2.JPEG]

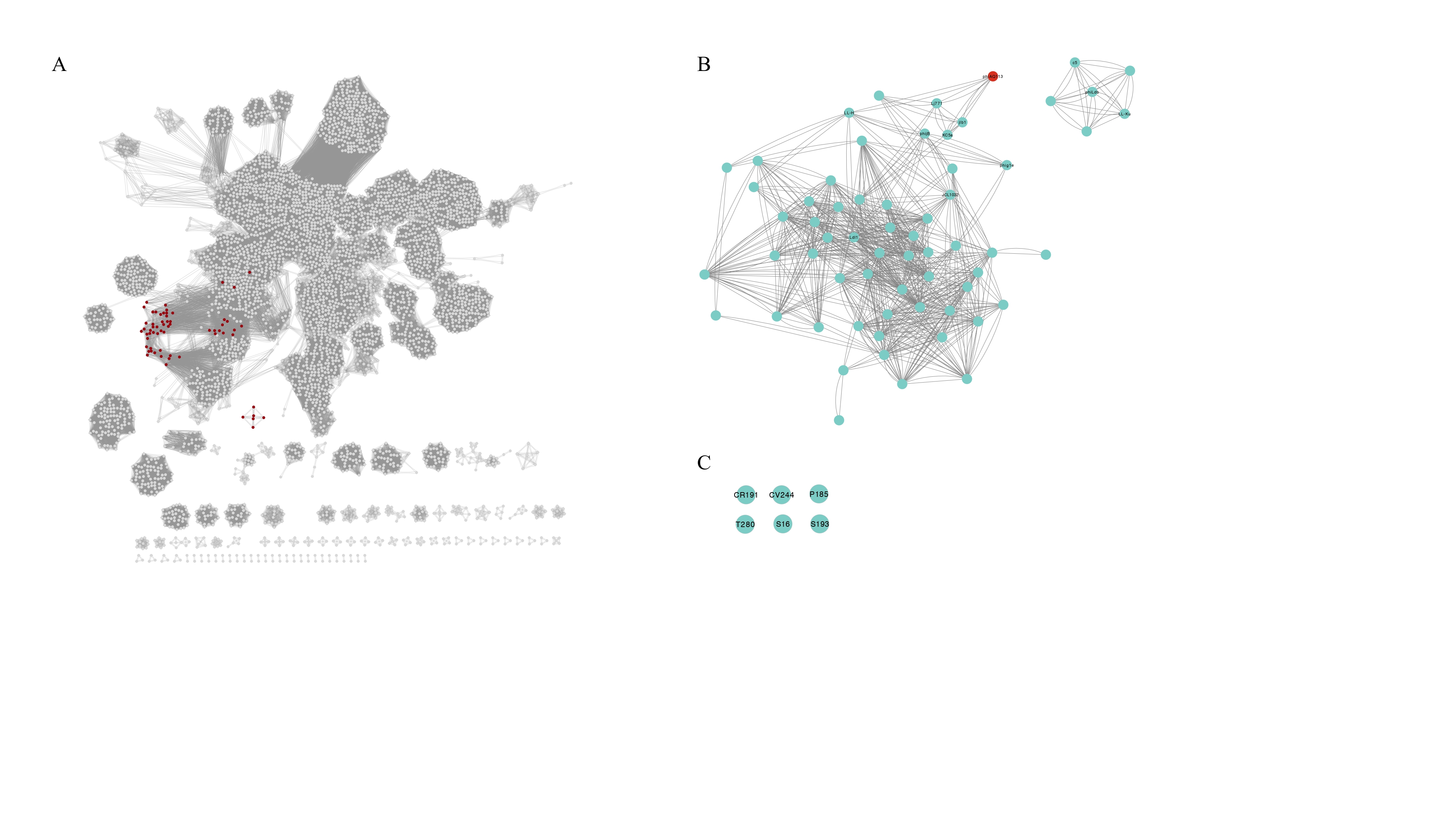

Supplement: SUPPLEMENTARY FIGURE S3 — Gene-sharing network analysis using vContact2. [file Image_3.JPEG]

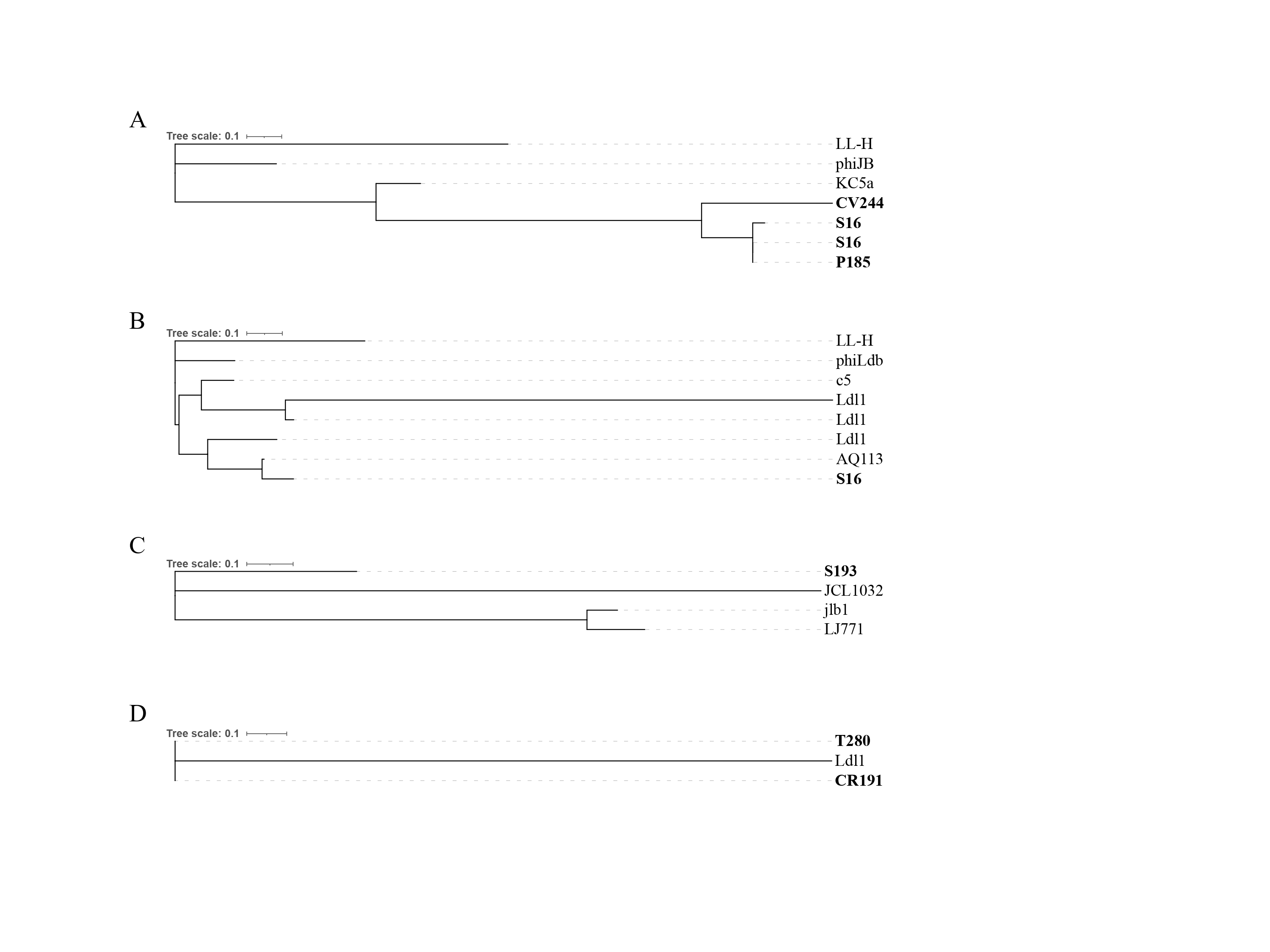

Supplement: SUPPLEMENTARY FIGURE S4 — Phylogenetic relationships of accessory regulatory proteins in Trentingrana prophages and related dairy Lactobacillus phages. [file Image_4.JPEG]
